# Phosphorus (P) use efficiency in rice is linked to tissue-specific biomass and P allocation patterns

**DOI:** 10.1038/s41598-020-61147-3

**Published:** 2020-03-09

**Authors:** Muhammad Irfan, Tariq Aziz, Muhammad Aamer Maqsood, Hafiz Muhammad Bilal, Kadambot H. M. Siddique, Minggang Xu

**Affiliations:** 1 Institute of Soil and Environmental Sciences, University of Agriculture, Faisalabad, 38040 Pakistan; 2 Soil and Environmental Sciences Division, Nuclear Institute of Agriculture, Tandojam, 70060 Pakistan; 3 The UWA Institute of Agriculture and UWA School of Agriculture and Environment, The University of Western Australia, Perth, WA 6001 Australia; 4 Department of Environmental Sciences, University of Okara, Okara, 56300 Pakistan; 5 National Engineering Laboratory for Improving Quality of Arable Land, Institute of Agri. Resources and Regional Planning, Chinese Academy of Agricultural Sciences, Beijing, 100081 China

**Keywords:** Plant physiology, Abiotic

## Abstract

Phosphorus (P) is a non-renewable resource which may be depleted within next few decades; hence high P use efficiency is need of time. Plants have evolved an array of adaptive mechanisms to enhance external P acquisition and reprioritize internal utilization under P deficiency. Tissue specific biomass and P allocation patterns may affect the P use efficiency in plants. six rice cultivars were grown in solution culture for 20 days and then were divided into two groups to receive either adequate P or no P that were harvested at 30, 40 and 50 days. Plants were dissected into various tissues/organs. Two rice cultivars viz Super Basmati (P-inefficient) and PS-2 (P-efficient) were grown in soil with no or 50 mg P kg^−1^ soil till maturity. Rice cultivars PS-2 and Basmati-2000 had higher P uptake, utilization efficiency and internal remobilization than other tested cultivars after P omission. Young leaves and roots were the major sinks while stems and mature leaves were the sources of P during P omission. In conclusion, biomass allocation and P accumulation among various tissues and P remobilization were major factors responsible for P efficiency.

## Introduction

Phosphorus (P) is the second-most essential element after nitrogen (N) for its impact on the productivity and health of aquatic and terrestrial ecosystems^1^. Being an essential element, P is considered as a major driver for optimum crop productivity on arable land around the globe^2–4^. However, suboptimal P availability on arable land forces researchers to determine the mechanisms for improving P acquisition and utilization, and to exploit these responses for the development of P-efficient cultivars^5,6^.

The identification of desirable crop plants for adaptation to low-P-input environments may enhance crop productivity and reduce the reliance on costly synthetic P fertilizers^6^. This approach is especially important for developing countries with limited resources. Under the current situation, farmers need P-efficient genotypes that perform better than other genotypes with equivalent P inputs^7–11^. Many crop plants have evolved morphological, physiological, biochemical and molecular adaptive systems to cope with P-deficiency stress, such as altered root architecture to explore more soil volume^12^, and increased carboxylate exudation containing phosphatases, nucleases and various organic acids^13,14^. These mechanisms and strategies are necessary to liberate or solubilize Pi from organic and other insoluble pools^15^, enhance Pi uptake capacity^16^, recycle internal Pi^13,17^, remobilize/retranslocate P from mature to young developing organs^18,19^, and reprioritize metabolic P utilization^20^. Cultivars with enhanced P efficiency could be an alternate strategy for overcoming the dilemma of P deficiency. Phosphorus efficiency can be divided into (i) P acquisition efficiency – the capacity of a cultivar to extract P from soil, and (ii) P utilization efficiency – the capacity of a cultivar to transform the acquired P into biomass/grain yield^21,22^.

The process of P remobilization from mature leaves frequently occurs during vegetative growth, when there is insufficient soil P available^23^. However, remobilization can also happen during the reproductive period, when new sinks are emerging while further P acquisition by plant roots is reduced^24^. The remobilization process during reproductive growth is generally associated with foliar senescence to ensure nutrient supply for developing tissues^25^ and phloem transport is mainly required for the remobilization process, as P is highly mobile in the phloem^26^. This mechanism of P remobilization is responsible for inter- and intra-specific differences in crop plants for P utilization. These traits are heritable and can be exploited to screen plants for high P-efficiency^27^.

Rice (*Oryza sativa* L.) is a major cereal crop known to provide calories to about one third of the population around the globe. According to estimates, global rice harvest removes P from fields of worth around $11 billion each year^28^. Different rice genotypes with higher P-use efficiency can be utilized in rice yield improvement programs. Enhanced internal P-utilization efficiency is required to complement higher P uptake traits for successful breeding of P efficient rice cultivars^29^. Screening of existing germplasm and identification of the responsible P-efficiency mechanism is needed to produce more P-efficient cultivars through conventional as well as molecular breeding ventures. The present study was conducted to identify P-efficient rice cultivars and evaluate the responsible mechanisms for P efficiency in two independent experiments.

## Results

### Experiment 1

#### Variation in growth response of rice cultivars under P starvation

The rice cultivars had significant (*P* ≤ 0.05) variations in plant height, root depth, root dry matter (RDM), shoot dry matter (SDM) and root:shoot ratio (RSR) under both adequate P (200 µM Pi) and P deficiency (Table 1). Plant height increased linearly with time at both P levels. Averaged across all harvests, PS-2 and Basmati-2000 had the tallest plants while Super Basmati had the shortest plants at both P levels. Rooting depth of all rice cultivars increased in response to P deficiency relative to adequate P supply. The cultivar PS-2 had the deepest roots at 39.6 cm and 42.8 cm with adequate P and no P, respectively, and KS-282 had the shallowest rooting depth at 27.4 cm with no P. The six rice cultivars varied in RDM with Basmati-2000 producing the maximum (0.53 g plant^−1^) and Super Basmati producing the minimum (0.20 g plant^−1^) amounts under P deficiency. The SDM of rice cultivars decreased significantly after exposure to P deficit, but the reduction varied significantly between cultivars. Overall, cultivar PS-2 produced the most SDM (3.31 g plant^−1^) under adequate P, while Basmati-2000 produced the most (1.36 g plant^−1^) with no P supplied. The RSR increased in all tested cultivars under P stress except for KS-282 which had no change at either P level. The maximum RSR after P omission (0.45) occurred in KSK-434 (Table 1).

**Table 1:** Plant height, root depth, root dry matter, shoot dry matter, and root:shoot ratio of six rice cultivars at 20, 30, 40, and 50 days after transplanting (DAT).

Cultivars	200 µM Pi				0 µM Pi		
	20 DAT	30 DAT	40 DAT	50 DAT	30 DAT	40 DAT	50 DAT
**Plant height (cm)**							
Basmati-2000	48.9 ± 0.91 ^cd^	62.3 ± 0.37^a^	64.8 ± 0.88^d^	77.5 ± 0.87^c^	60.2 ± 0.60^a^	67.4 ± 0.72^b^	84.0 ± 2.58^a^
Super Basmati	46.8 ± 0.95^d^	51.3 ± 0.88^c^	61.1 ± 0.83^e^	75.7 ± 0.44 ^cd^	50.3 ± 0.67^d^	57.4 ± 0.73^d^	66.4 ± 0.86^d^
PS-2	58.0 ± 0.70^a^	61.8 ± 0.88^a^	83.2 ± 0.43^a^	90.8 ± 0.62^a^	59.7 ± 0.44^a^	73.2 ± 2.62^a^	76.0 ± 0.76^b^
KSK-434	50.8 ± 0.68^bc^	60.5 ± 0.87^ab^	64.1 ± 0.47^d^	77.7 ± 0.64^c^	56.0 ± 0.58^b^	66.1 ± 0.75^b^	70.0 ± 0.58^c^
KS-282	51.6 ± 0.90^b^	58.7 ± 0.88^b^	75.6 ± 0.78^b^	81.0 ± 0.76^b^	56.7 ± 0.67^b^	71.7 ± 0.84^a^	77.0 ± 1.87^b^
IR-6	51.5 ± 0.77^b^	59.2 ± 0.73^b^	67.7 ± 0.94^c^	74.8 ± 0.79^d^	53.8 ± 0.15^c^	61.6 ± 0.72^c^	63.7 ± 0.73^e^
LSD_0.05_	2.6	2.4	2.3	2.2	1.7	3.9	2.3
**Root depth (cm)**							
Basmati-2000	21.7 ± 0.54^d^	29.0 ± 0.58^bc^	31.3 ± 1.62^b^	32.7 ± 0.93^bc^	28.2 ± 0.60^b^	31.1 ± 0.96^c^	42.3 ± 0.44^bc^
Super Basmati	24.5 ± 0.33^c^	28.0 ± 2.52^bcd^	31.5 ± 3.13^b^	33.2 ± 2.01b^c^	26.3 ± 0.77^bc^	28.0 ± 0.66^d^	41.3 ± 0.44^c^
PS-2	33.3 ± 0.59^a^	37.7 ± 0.88^a^	43.3 ± 0.71^a^	44.0 ± 2.86^a^	33.7 ± 0.88^a^	40.7 ± 0.64^a^	54.0 ± 0.58^a^
KSK-434	30.5 ± 0.66^b^	33.3 ± 3.09^ab^	34.5 ± 0.29^b^	35.5 ± 2.80^b^	33.2 ± 0.73^a^	36.3 ± 0.79^b^	44.1 ± 0.75^b^
KS-282	22.1 ± 0.55^d^	23.2 ± 0.44 ^cd^	24.1 ± 0.47^c^	25.3 ± 1.70^d^	25.3 ± 0.88^c^	29.3 ± 0.97 ^cd^	27.7 ± 0.88^d^
IR-6	19.4 ± 0.41^e^	22.8 ± 2.09^d^	23.9 ± 1.30^c^	27.2 ± 2.92 ^cd^	24.7 ± 0.92^c^	31.1 ± 0.67^c^	28.2 ± 0.60^d^
LSD_0.05_	1.6	4.8	4.9	6.1	2.5	2.4	1.9
**Root dry matter (g plant**^−**1**^**)**							
Basmati-2000	0.30 ± 0.03^a^	0.40 ± 0.01 ^cd^	0.42 ± 0.05^c^	0.79 ± 0.01^c^	0.48 ± 0.03^a^	0.52 ± 0.09^a^	0.58 ± 0.01^a^
Super Basmati	0.16 ± 0.04^b^	0.48 ± 0.01^b^	0.47 ± 0.12^c^	0.65 ± 0.02 ^cd^	0.17 ± 0.03^c^	0.20 ± 0.09^d^	0.23 ± 0.07^c^
PS-2	0.37 ± 0.05^a^	0.50 ± 0.02^b^	1.01 ± 0.22^a^	1.26 ± 0.12^ab^	0.39 ± 0.04^ab^	0.45 ± 0.12^b^	0.49 ± 0.15^ab^
KSK-434	0.33 ± 0.04^a^	0.88 ± 0.01^a^	1.02 ± 0.15^a^	1.13 ± 0.03^b^	0.35 ± 0.09^b^	0.38 ± 0.02^c^	0.39 ± 0.04^b^
KS-282	0.20 ± 0.10^b^	0.42 ± 0.02^c^	0.71 ± 0.08^b^	1.42 ± 0.07^a^	0.22 ± 0.06^c^	0.23 ± 0.11^d^	0.20 ± 0.05^c^
IR-6	0.17 ± 0.02^b^	0.38 ± 0.01^d^	0.54 ± 0.11^c^	0.47 ± 0.04^d^	0.19 ± 0.04^c^	0.21 ± 0.05^d^	0.23 ± 0.08^c^
LSD_0.05_	0.13	0.04	0.21	0.19	0.12	0.07	0.18
**Shoot dry matter (g plant**^**−1**^**)**							
Basmati-2000	1.13 ± 0.01^a^	1.75 ± 0.01^bc^	1.73 ± 0.18^d^	4.16 ± 0.45^d^	1.14 ± 0.02^a^	1.38 ± 0.14^a^	0.84 ± 0.10^c^
Super Basmati	0.51 ± 0.07 ^cd^	1.68 ± 0.07^bc^	2.36 ± 0.86^c^	4.36 ± 0.21^d^	0.55 ± 0.01 ^cd^	0.60 ± 0.09^c^	0.53 ± 0.05^d^
PS-2	0.94 ± 0.19^b^	1.82 ± 0.10^b^	4.29 ± 0.52^a^	6.19 ± 0.65^a^	1.02 ± 0.06^a^	1.41 ± 0.06^a^	1.33 ± 0.06^a^
KSK-434	0.66 ± 0.09^c^	2.85 ± 0.05^a^	4.45 ± 0.55^a^	4.71 ± 0.50 ^cd^	0.72 ± 0.06^b^	0.92 ± 0.04^b^	0.91 ± 0.03^b^
KS-282	0.59 ± 0.11 ^cd^	1.62 ± 0.08^c^	3.74 ± 0.43^b^	5.72 ± 0.93^b^	0.68 ± 0.12^bc^	0.87 ± 0.05^b^	0.82 ± 0.04^c^
IR-6	0.47 ± 0.09^d^	1.77 ± 0.03^bc^	2.56 ± 0.54^c^	4.66 ± 0.21 ^cd^	0.51 ± 0.15^d^	0.53 ± 0.07^c^	0.60 ± 0.07^bc^
LSD_0.05_	0.19	0.16	0.90	0.69	0.20	0.26	0.26
**Root:shoot ratio**							
Basmati-2000	0.27 ± 0.02^d^	0.23 ± 0.01 ^cd^	0.14 ± 0.04^b^	0.19 ± 0.02^ab^	0.42 ± 0.02^ab^	0.38 ± 0.03^ab^	0.69 ± 0.05^a^
Super Basmati	0.31 ± 0.04 ^cd^	0.29 ± 0.01^ab^	0.19 ± 0.04^ab^	0.15 ± 0.01^bc^	0.31 ± 0.06^b^	0.40 ± 0.13^ab^	0.43 ± 0.13^b^
PS-2	0.45 ± 0.16^ab^	0.28 ± 0.02^ab^	0.24 ± 0.01^a^	0.21 ± 0.04^ab^	0.38 ± 0.05^ab^	0.33 ± 0.09^ab^	0.37 ± 0.11^c^
KSK-434	0.53 ± 0.09^a^	0.31 ± 0.01^a^	0.23 ± 0.01^a^	0.24 ± 0.02^a^	0.48 ± 0.11^a^	0.42 ± 0.03^a^	0.43 ± 0.06^b^
KS-282	0.36 ± 0.18 ^cd^	0.27 ± 0.02^bc^	0.19 ± 0.01^ab^	0.26 ± 0.05^a^	0.30 ± 0.10^b^	0.29 ± 0.05^b^	0.24 ± 0.05^d^
IR-6	0.40 ± 0.09^bc^	0.21 ± 0.01^d^	0.21 ± 0.02^a^	0.10 ± 0.01^c^	0.50 ± 0.09^a^	0.40 ± 0.11^ab^	0.38 ± 0.10^c^
LSD_0.05_	0.12	0.04	0.06	0.08	0.17	0.12	0.16

Initially, plants were grown for 20 days with adequate P (200 µM Pi). At 20 DAT, three replications of each cultivar were harvested, and the remaining plants were divided into two groups receiving adequate P or no P and harvested at 30, 40, and 50 DAT. Data are shown as means ± standard error (mean ± SE, n = 3). Means sharing identical letter(s) in the same column indicate non-significant differences among cultivars at each harvest (LSD test, *P* ≤ 0.05).

#### Genotype-dependent variation in biomass partitioning

The data for dry matter production of various plant tissues, i.e., stem, young leaves (tip and base), and mature leaves (tip and base) growing with or without P is presented in Table 2. Interestingly, stem dry matter declined significantly with P stress in all rice cultivars. With adequate P, rice cultivars PS-2 and Super Basmati produced the maximum (1.49 g plant^−1^) and minimum (0.97 g plant^−1^) stem dry matter, respectively, while the corresponding values under P stress were 0.44 g plant^−1^ in Basmati-2000 and 0.14 g plant^−1^ in IR-6. Overall, KSK-434 produced the most biomass in the young leaf tip (0.48 g plant^−1^) and young leaf base (0.53 g plant^−1^) under adequate P supply, while Basmati-2000 produced the maximum biomass (0.20 and 0.25 g plant^−1^, respectively) after P omission. The cultivar PS-2 accumulated the most biomass in the mature leaf tip (0.27 g plant^−1^) and mature leaf base (0.29 g plant^−1^), and IR-6 produced the least biomass (0.11 g plant^−1^ and 0.07 g plant^−1^, respectively) after P omission. However, under adequate P supply, cultivars KSK-434 and Basmati-2000 produced the maximum (0.40 and 0.44 g plant^−1^) and minimum (0.25 and 0.28 g plant^−1^) biomass in the mature leaf tip and mature leaf base, respectively. Overall, Basmati-2000 produced the most dry matter in young leaves (tip + base), and PS-2 produced the most in mature leaves (tip + base) under P deficiency.

**Table 2:** Dry matter production of various plant tissues (stem, young leaf tip, young leaf base, mature leaf tip, and mature leaf base) of six rice cultivars at 20, 30, 40, and 50 days after transplanting (DAT).

Cultivars	200 µM Pi				0 µM Pi		
	20 DAT	30 DAT	40 DAT	50 DAT	30 DAT	40 DAT	50 DAT
**Stem (g plant**^−**1**^**)**							
Basmati-2000	0.25 ± 0.02^ab^	0.95 ± 0.08^ab^	0.79 ± 0.13^c^	2.09 ± 0.26^b^	0.35 ± 0.04^a^	0.46 ± 0.03^a^	0.52 ± 0.11^a^
Super Basmati	0.15 ± 0.03^c^	0.86 ± 0.27^b^	0.99 ± 0.34^c^	1.86 ± 0.10^b^	0.17 ± 0.02^b^	0.17 ± 0.06^b^	0.15 ± 0.04^c^
PS-2	0.32 ± 0.08^a^	0.96 ± 0.10^ab^	2.01 ± 0.22^a^	2.66 ± 0.17^a^	0.33 ± 0.09^a^	0.37 ± 0.02^a^	0.33 ± 0.03^b^
KSK-434	0.20 ± 0.04^abc^	1.38 ± 0.32^a^	1.72 ± 0.25^ab^	1.98 ± 0.20^b^	0.21 ± 0.04^b^	0.25 ± 0.03^b^	0.19 ± 0.07^c^
KS-282	0.19 ± 0.09^bc^	0.81 ± 0.22^b^	1.53 ± 0.18^b^	2.60 ± 0.23^a^	0.16 ± 0.03^b^	0.19 ± 0.05^b^	0.17 ± 0.03^c^
IR-6	0.16 ± 0.04^bc^	0.91 ± 0.20^b^	1.09 ± 0.19^c^	2.11 ± 0.09^b^	0.17 ± 0.02^b^	0.13 ± 0.04^c^	0.10 ± 0.03^d^
LSD_0.05_	0.11	0.36	0.43	0.48	0.12	0.06	0.08
**Young leaf tip (g plant**^−**1**^**)**							
Basmati-2000	0.22 ± 0.02^a^	0.17 ± 0.03^b^	0.25 ± 0.03^e^	0.49 ± 0.06^c^	0.17 ± 0.05^a^	0.19 ± 0.02^a^	0.23 ± 0.03^ab^
Super Basmati	0.07 ± 0.02^bc^	0.17 ± 0.10^b^	0.40 ± 0.15^d^	0.69 ± 0.04^b^	0.07 ± 0.01 ^cd^	0.10 ± 0.01^b^	0.15 ± 0.04^b^
PS-2	0.10 ± 0.03^b^	0.16 ± 0.02^b^	0.67 ± 0.15^ab^	0.97 ± 0.25^a^	0.12 ± 0.02^b^	0.21 ± 0.03^a^	0.26 ± 0.05^a^
KSK-434	0.08 ± 0.01^b^	0.31 ± 0.08^a^	0.82 ± 0.06^a^	0.72 ± 0.12^b^	0.09 ± 0.03^bc^	0.16 ± 0.02^ab^	0.22 ± 0.02^ab^
KS-282	0.03 ± 0.01^c^	0.17 ± 0.06^b^	0.57 ± 0.11^c^	0.88 ± 0.27^ab^	0.09 ± 0.01^bcd^	0.21 ± 0.09^a^	0.26 ± 0.01^a^
IR-6	0.04 ± 0.01^c^	0.18 ± 0.06^b^	0.38 ± 0.10^d^	0.69 ± 0.16^b^	0.05 ± 0.02^d^	0.11 ± 0.03^b^	0.15 ± 0.04^b^
LSD_0.05_	0.04	0.14	0.13	0.12	0.04	0.09	0.10
**Young leaf base (g plant**^**−1**^**)**							
Basmati-2000	0.14 ± 0.02^a^	0.23 ± 0.03^b^	0.27 ± 0.03^d^	0.56 ± 0.05^c^	0.21 ± 0.05^a^	0.25 ± 0.03^a^	0.28 ± 0.03^a^
Super Basmati	0.09 ± 0.02^ab^	0.23 ± 0.10^b^	0.42 ± 0.15^c^	0.76 ± 0.10^b^	0.10 ± 0.02^bc^	0.14 ± 0.01^b^	0.11 ± 0.01^b^
PS-2	0.12 ± 0.03^a^	0.22 ± 0.02^b^	0.69 ± 0.15^b^	1.04 ± 0.25^a^	0.13 ± 0.02^bc^	0.18 ± 0.02^ab^	0.14 ± 0.02^b^
KSK-434	0.10 ± 0.01^ab^	0.37 ± 0.06^a^	0.84 ± 0.06^a^	0.79 ± 0.12^b^	0.14 ± 0.02^b^	0.16 ± 0.03^b^	0.15 ± 0.02^b^
KS-282	0.05 ± 0.01^b^	0.23 ± 0.041^b^	0.59 ± 0.11^b^	0.95 ± 0.19^ab^	0.08 ± 0.03^c^	0.15 ± 0.04^b^	0.13 ± 0.01^b^
IR-6	0.06 ± 0.01^b^	0.24 ± 0.06^b^	0.40 ± 0.10 ^cd^	0.76 ± 0.11^b^	0.09 ± 0.03^bc^	0.12 ± 0.02^b^	0.13 ± 0.06^b^
LSD_0.05_	0.05	0.12	0.13	0.19	0.06	0.08	0.10
**Mature leaf tip (g plant**^−**1**^**)**							
Basmati-2000	0.14 ± 0.02^b^	0.17 ± 0.01^b^	0.19 ± 0.01^c^	0.49 ± 0.06^c^	0.18 ± 0.01^a^	0.21 ± 0.03^ab^	0.26 ± 0.02^b^
Super Basmati	0.08 ± 0.01^c^	0.18 ± 0.07^b^	0.25 ± 0.12^bc^	0.51 ± 0.04^c^	0.08 ± 0.02^c^	0.09 ± 0.01^d^	0.11 ± 0.02^d^
PS-2	0.16 ± 0.05^a^	0.20 ± 0.03^b^	0.45 ± 0.02^a^	0.75 ± 0.12^a^	0.19 ± 0.02^a^	0.28 ± 0.08^a^	0.34 ± 0.06^a^
KSK-434	0.11 ± 0.02^bc^	0.36 ± 0.09^a^	0.52 ± 0.09^a^	0.59 ± 0.04^bc^	0.15 ± 0.03^ab^	0.19 ± 0.03^abc^	0.21 ± 0.03^bc^
KS-282	0.13 ± 0.02^b^	0.17 ± 0.02^b^	0.50 ± 0.09^a^	0.63 ± 0.10^b^	0.17 ± 0.06^a^	0.16 ± 0.01^bc^	0.17 ± 0.01 ^cd^
IR-6	0.09 ± 0.02^c^	0.19 ± 0.06^b^	0.33 ± 0.08^b^	0.54 ± 0.07^c^	0.10 ± 0.06^bc^	0.11 ± 0.03^c^	0.13 ± 0.02^d^
LSD_0.05_	0.03	0.14	0.12	0.10	0.07	0.12	0.10
**Mature leaf base (g plant**^−**1**^**)**							
Basmati-2000	0.16 ± 0.03^ab^	0.23 ± 0.01^b^	0.23 ± 0.02^c^	0.52 ± 0.04^c^	0.24 ± 0.01^a^	0.27 ± 0.04^b^	0.26 ± 0.02^a^
Super Basmati	0.12 ± 0.04^b^	0.24 ± 0.06^b^	0.29 ± 0.10^bc^	0.54 ± 0.04^c^	0.13 ± 0.02^c^	0.11 ± 0.01 ^cd^	0.05 ± 0.01^c^
PS-2	0.23 ± 0.08^a^	0.26 ± 0.03^b^	0.49 ± 0.04^a^	0.78 ± 0.12^a^	0.25 ± 0.01^a^	0.36 ± 0.03^a^	0.25 ± 0.02^a^
KSK-434	0.15 ± 0.02^ab^	0.42 ± 0.09^a^	0.56 ± 0.11^a^	0.62 ± 0.04^b^	0.13 ± 0.03^c^	0.16 ± 0.03^c^	0.14 ± 0.03^b^
KS-282	0.17 ± 0.02^ab^	0.23 ± 0.02^b^	0.54 ± 0.09^a^	0.66 ± 0.11^b^	0.18 ± 0.05^b^	0.15 ± 0.03^c^	0.10 ± 0.02^bc^
IR-6	0.13 ± 0.02^b^	0.25 ± 0.06^b^	0.37 ± 0.08^b^	0.57 ± 0.04^c^	0.09 ± 0.01^c^	0.07 ± 0.01^d^	0.05 ± 0.01^c^
LSD_0.05_	0.08	0.15	0.14	0.11	0.05	0.08	0.06

Initially, plants were grown for 20 days with adequate P (200 µM Pi). At 20 DAT, three replications of each cultivar were harvested, and the remaining plants were divided into two groups receiving adequate P or no P and harvested at 30, 40, and 50 DAT. Data are shown as means ± standard error (mean ± SE, n = 3). Means sharing identical letter(s) in the same column indicate non-significant differences among cultivars at each harvest (LSD test, *P* ≤ 0.05).

#### P acquisition and tissue-specific Pi allocation

The P concentration [P] in different plant tissues, i.e., root, stem, young leaves (tip and base), and mature leaves (tip and base) at 20, 30, 40 and 50 DAT at each P level is given in Table 3. The [P] in plant roots with no P added increased from 20 to 50 DAT. Overall, PS-2 had the highest root [P] with 4.47 and 3.13 mg g^−1^ and IR-6 had the least [P] with 2.40 and 1.66 mg g^−1^ under adequate and P stress, respectively. Stem [P] decreased with time in all cultivars under P deficiency. Under P stress, KS-282 had the highest stem [P] (2.73 mg g^−1^) while Basmati-2000 had the least (1.61 mg g^−1^). The six rice cultivars varied for [P] in young leaf tips and basal sections at both P levels. After P omission, PS-2 had the highest [P] with 3.18 mg g^−1^ in young leaf tips while Basmati-2000 had the least [P] with 2.06 mg g^−1^. The rice cultivars KS-282 and Super Basmati had the highest (3.23 mg g^−1^) and lowest (2.22 mg g^−1^) [P] in the young leaf base, respectively, among the tested cultivars under P deficiency. The [P] in mature leaf tips and mature leaf base was higher in IR-6 and KSK-434 (2.36 and 1.73 mg g^−1^, respectively) after P omission.

**Table 3:** Phosphorus concentration [P] of various plant tissues (root, stem, young leaf tip, young leaf base, mature leaf tip, and mature leaf base) of six rice cultivars at 20, 30, 40, and 50 days after transplanting (DAT).

Cultivars	200 µM Pi				0 µM Pi		
	20 DAT	30 DAT	40 DAT	50 DAT	30 DAT	40 DAT	50 DAT
**Root (mg g**^−**1**^**)**							
Basmati-2000	2.47 ± 0.21^b^	4.16 ± 0.30^a^	4.20 ± 0.28^a^	5.97 ± 0.19^a^	2.35 ± 0.54^b^	2.42 ± 0.04^b^	2.51 ± 0.16^b^
Super Basmati	1.66 ± 0.32 ^cd^	3.26 ± 0.15^b^	3.99 ± 0.36^ab^	4.28 ± 0.15^bc^	1.75 ± 0.17^bc^	1.79 ± 0.15^c^	1.93 ± 0.25^c^
PS-2	3.05 ± 0.54^a^	4.59 ± 0.21^a^	4.29 ± 0.45^a^	5.94 ± 0.55^a^	3.02 ± 0.16^a^	3.15 ± 0.13^a^	3.23 ± 0.08^a^
KSK-434	1.75 ± 0.22^c^	3.27 ± 0.14^b^	4.03 ± 0.23^ab^	4.67 ± 0.27^b^	1.92 ± 0.32^bc^	2.46 ± 0.15^b^	2.62 ± 0.25^b^
KS-282	1.86 ± 0.29^c^	2.80 ± 0.27^b^	3.97 ± 0.36^ab^	4.16 ± 0.08^c^	1.89 ± 0.35^bc^	1.99 ± 0.26^c^	1.99 ± 0.46^c^
IR-6	1.34 ± 0.11^d^	1.56 ± 0.16^c^	3.57^ ± ^0.18^c^	3.14 ± 0.03^d^	1.47 ± 0.25^c^	1.69 ± 0.24^c^	1.83 ± 0.31^c^
LSD_0.05_	0.36	0.66	0.70	0.43	0.82	0.43	0.61
**Stem (mg g**^−**1**^**)**							
Basmati-2000	2.91 ± 0.25^c^	2.56 ± 0.83^d^	3.87 ± 0.39^abc^	4.16 ± 0.44^c^	2.01 ± 0.26^b^	1.64 ± 0.19^b^	1.17 ± 0.02^b^
Super Basmati	2.05 ± 0.13^d^	2.82 ± 0.31^d^	3.55 ± 0.14^c^	3.23 ± 0.29^d^	1.74 ± 0.13^b^	1.78 ± 0.26^ab^	1.47 ± 0.23^b^
PS-2	4.03 ± 0.14^ab^	5.82 ± 0.56^ab^	3.81 ± 0.53^bc^	5.01 ± 0.09^ab^	3.83 ± 0.13^a^	1.48 ± 0.09^b^	1.32 ± 0.26^b^
KSK-434	4.56 ± 0.11^a^	6.52 ± 0.50^a^	3.94 ± 0.12^abc^	5.39 ± 0.71^a^	3.97 ± 0.23^a^	1.59 ± 0.13^b^	1.51 ± 0.40^b^
KS-282	3.90 ± 0.27^b^	4.47 ± 0.78^bc^	4.43 ± 0.15^a^	4.55 ± 0.59^bc^	3.78 ± 0.24^a^	2.25 ± 0.36^a^	2.17 ± 0.39^a^
IR-6	2.01 ± 0.15^d^	3.63 ± 0.34 ^cd^	4.21 ± 0.30^ab^	4.49 ± 0.18^bc^	1.93 ± 0.33^b^	1.81 ± 0.57^ab^	1.55 ± 0.26^b^
LSD_0.05_	0.57	1.87	0.64	0.86	0.88	0.56	0.62
**Young leaf tip (mg g**^−**1**^**)**							
Basmati-2000	2.53 ± 0.05^ab^	3.49 ± 0.41^bc^	3.49 ± 0.10^ab^	3.44 ± 0.51^c^	1.99 ± 0.13^b^	2.04 ± 0.19^c^	2.16 ± 0.04^c^
Super Basmati	1.75 ± 0.15^c^	2.98 ± 0.12^c^	3.11 ± 0.21^bc^	2.52 ± 0.20^d^	2.56 ± 0.51^ab^	2.57 ± 0.12^b^	2.63 ± 0.34^bc^
PS-2	2.69 ± 0.12^a^	4.72 ± 0.37^a^	3.75 ± 0.44^a^	4.18 ± 0.51^b^	2.77 ± 0.21^a^	3.33 ± 0.53^a^	3.45 ± 0.28^a^
KSK-434	2.58 ± 0.24^a^	3.74 ± 0.26^bc^	3.75 ± 0.49^a^	5.38 ± 0.21^a^	2.57 ± 0.26^ab^	2.70 ± 0.26^b^	3.15 ± 0.37^a^
KS-282	2.44 ± 0.16^ab^	4.34 ± 0.32^ab^	4.01 ± 0.30^a^	4.57 ± 0.54^b^	2.81 ± 0.30^a^	2.98 ± 0.60^ab^	3.01 ± 0.09^ab^
IR-6	1.91 ± 0.10^bc^	3.68 ± 0.47^bc^	2.75 ± 0.24^c^	2.93 ± 0.46 ^cd^	2.02 ± 0.24^b^	2.19 ± 0.41^c^	2.23 ± 0.57^c^
LSD_0.05_	0.67	0.96	0.61	0.52	0.71	0.32	0.53
**Young leaf base (mg g**^−**1**^**)**							
Basmati-2000	3.17 ± 0.12^ab^	3.83 ± 0.19^c^	4.24 ± 0.69^a^	4.03 ± 0.48^a^	3.43 ± 0.15^a^	2.95 ± 0.13^b^	2.96 ± 0.06^a^
Super Basmati	3.24 ± 0.21^ab^	5.19 ± 0.35^b^	3.98 ± 0.47^ab^	3.59 ± 0.15^ab^	1.98 ± 0.45^c^	2.40 ± 0.29^c^	2.28 ± 0.07^b^
PS-2	3.31 ± 0.20^ab^	3.66 ± 0.28^c^	3.45 ± 0.12^ab^	3.46 ± 0.19^ab^	2.84 ± 0.40^b^	3.04 ± 0.07^ab^	2.94 ± 0.12^a^
KSK-434	3.66 ± 0.05^a^	6.14 ± 0.48^a^	3.41 ± 0.51^bc^	3.05 ± 0.25^b^	3.19 ± 0.59^ab^	3.17 ± 0.11^ab^	2.96 ± 0.41^a^
KS-282	3.39 ± 0.17^ab^	3.99 ± 0.42^c^	2.58 ± 0.33^c^	2.37 ± 0.27^c^	3.59 ± 0.26^a^	3.43 ± 0.43^a^	2.67 ± 0.39^ab^
IR-6	3.13 ± 0.17^b^	5.52 ± 0.18^b^	3.43 ± 0.10^ab^	3.74 ± 0.63^a^	3.24 ± 0.12^ab^	3.20 ± 0.25^ab^	2.83 ± 0.55^ab^
LSD_0.05_	0.50	0.62	0.83	0.65	0.57	0.47	0.63
**Mature leaf tip (mg g**^−**1**^**)**							
Basmati-2000	3.42 ± 0.26^a^	3.10 ± 0.26^c^	2.32 ± 0.27^c^	2.86 ± 0.28^bc^	2.70 ± 0.27^ab^	2.27 ± 0.28^a^	1.38 ± 0.07^b^
Super Basmati	1.99 ± 0.19^d^	5.65 ± 0.82^a^	3.15 ± 0.15^a^	3.69 ± 0.35^a^	2.09 ± 0.12^b^	1.56 ± 0.13^bc^	1.60 ± 0.10^ab^
PS-2	2.50 ± 0.40^bcd^	3.38 ± 0.70^c^	2.65 ± 0.03^bc^	2.77 ± 0.26^bc^	2.80 ± 0.35^ab^	1.49 ± 0.26^bc^	1.52 ± 0.24^b^
KSK-434	3.08 ± 0.09^ab^	4.45 ± 0.83^b^	3.13 ± 0.13^a^	3.51 ± 0.24^ab^	3.23 ± 0.33^a^	1.99 ± 0.09^ab^	1.60 ± 0.20^ab^
KS-282	2.21 ± 0.34 ^cd^	3.54 ± 0.10^c^	2.77 ± 0.64^ab^	3.04 ± 0.13^abc^	2.29 ± 0.38^b^	1.38 ± 0.19^c^	1.44 ± 0.08^b^
IR-6	2.91 ± 0.10^abc^	4.13 ± 0.25^b^	2.64 ± 0.28^bc^	2.74 ± 0.30^c^	3.24 ± 0.45^a^	1.75 ± 0.35^abc^	2.08 ± 0.51^a^
LSD_0.05_	0.79	0.87	0.44	0.72	0.81	0.59	0.55
**Mature leaf base (mg g**^**−1**^**)**							
Basmati-2000	2.16 ± 0.18^a^	3.34 ± 0.11 ^cd^	2.52 ± 0.64^c^	2.27 ± 0.09^d^	2.40 ± 0.48^a^	1.38 ± 0.18^ab^	1.07 ± 0.03^b^
Super Basmati	1.53 ± 0.17^bc^	4.98 ± 0.46^a^	3.77 ± 0.14^b^	4.30 ± 0.42^a^	1.77 ± 0.16^b^	1.53 ± 0.15^a^	1.36 ± 0.06^a^
PS-2	1.63 ± 0.33^bc^	3.76 ± 0.07^bc^	3.52 ± 0.13^b^	3.68 ± 0.43^b^	1.68 ± 0.20^b^	1.40 ± 0.14^a^	1.19 ± 0.13^ab^
KSK-434	2.20 ± 0.27^a^	4.88 ± 0.78^a^	4.31 ± 0.52^a^	4.54 ± 0.32^a^	2.30 ± 0.11^a^	1.48 ± 0.11^a^	1.39 ± 0.12^a^
KS-282	1.97 ± 0.18^ab^	2.87 ± 0.33^d^	2.81 ± 0.27^c^	3.07 ± 0.20^c^	2.24 ± 0.14^a^	1.44 ± 0.24^a^	1.18 ± 0.04^ab^
IR-6	1.73 ± 0.23^bc^	3.97 ± 0.10^b^	2.76 ± 0.36^c^	2.82 ± 0.07^c^	1.36 ± 0.15^b^	1.22 ± 0.18^b^	1.56 ± 0.24^a^
LSD_0.05_	0.42	0.62	0.41	0.59	0.47	0.15	0.29

Initially, plants were grown for 20 days with adequate P (200 µM Pi). At 20 DAT, three replications of each cultivar were harvested, and the remaining plants were divided into two groups receiving adequate P or no P and harvested at 30, 40, and 50 DAT. Data are shown as means ± standard error (mean ± SE, n = 3). Means sharing identical letter(s) in the same column indicate non-significant differences among cultivars at each harvest (LSD test, *P* ≤ 0.05).

The cultivars varied significantly (*P* ≤ 0.05) in P uptake in different plant parts when grown under adequate P or P stress (Table 4). The highest root P uptake under adequate P or P stress was in PS-2 with 3.77 and 1.38 mg plant^−1^, respectively. Likewise, PS-2 had higher stem P uptake at adequate P supply and after P stress (6.96 mg plant^−1^ and 0.73 mg plant^−1^, respectively). The maximum P uptake (0.63 mg plant^−1^) by young leaf tips occurred in PS-2 while the minimum (0.21 mg plant^−1^) occurred in IR-6 under P stress. Basmati-2000 and Super Basmati had the highest (0.76 mg plant^−1^) and lowest (0.26 mg plant^−1^) P uptake by the young leaf base after P omission. Mature leaf tips accumulated higher P contents than the mature leaf base after P omission. PS-2 had the highest P uptake by the mature tip and basal sections (0.49 and 0.41 mg plant^−1^, respectively) during P stress. When comparing P uptake between young and mature leaves, young leaves had higher P uptake at adequate P and under P stress. Averaged across all harvests, PS-2 had the highest total P uptake (4.10 mg plant^−1^) while IR-6 had the lowest (1.35 mg plant^−1^) under P stress.

**Table 4:** Phosphorus uptake (mg plant^−1^) by various plant tissues (root, stem, young leaf tip, young leaf base, mature leaf tip, mature leaf base, and whole plant) in six rice cultivars at 20, 30, 40, and 50 days after transplanting (DAT).

Cultivars	200 µM Pi				0 µM Pi		
	20 DAT	30 DAT	40 DAT	50 DAT	30 DAT	40 DAT	50 DAT
**Root (mg plant**^−**1**^**)**							
Basmati-2000	0.76 ± 0.12^b^	1.68 ± 0.16^c^	0.96 ± 0.14^d^	4.72 ± 0.15^d^	1.11 ± 0.25^a^	1.27 ± 0.23^a^	1.45 ± 0.11^a^
Super Basmati	0.29 ± 0.10^de^	1.57 ± 0.06^c^	1.97 ± 0.21^c^	2.79 ± 0.16^e^	0.30 ± 0.06^c^	0.31 ± 0.11^c^	0.42 ± 0.09^c^
PS-2	1.17 ± 0.34^a^	2.29 ± 0.05^b^	4.21 ± 0.44^a^	7.41 ± 0.64^a^	1.16 ± 0.10^a^	1.40 ± 0.33^a^	1.58 ± 0.48^a^
KSK-434	0.60 ± 0.15^c^	2.89 ± 0.14^a^	4.05 ± 0.13^a^	5.25 ± 0.19^c^	0.61 ± 0.06^b^	0.95 ± 0.08^b^	1.01 ± 0.02^b^
KS-282	0.41 ± 0.26^d^	1.19 ± 0.15^d^	2.77 ± 0.31^b^	5.92 ± 0.18^b^	0.34 ± 0.11^c^	0.37 ± 0.12^c^	0.37 ± 0.09^c^
IR-6	0.24 ± 0.04^e^	0.59 ± 0.05^e^	1.89 ± 0.11^c^	1.48 ± 0.12 ^f^	0.29 ± 0.10^c^	0.31 ± 0.02^c^	0.32 ± 0.04^c^
LSD_0.05_	0.15	0.22	0.52	0.53	0.16	0.13	0.36
**Stem (mg plant**^−**1**^**)**							
Basmati-2000	0.74 ± 0.11^b^	2.28 ± 0.73^d^	3.02 ± 0.52^c^	8.52 ± 0.69^d^	0.75 ± 0.28^b^	0.76 ± 0.13^a^	0.60 ± 0.12^a^
Super Basmati	0.32 ± 0.07^c^	2.60 ± 0.96^d^	3.60 ± 0.29^bc^	6.01 ± 0.64^e^	0.29 ± 0.05^d^	0.27 ± 0.08^d^	0.20 ± 0.04^c^
PS-2	1.33 ± 0.28^a^	5.66 ± 1.02^c^	7.54 ± 0.95^a^	13.32 ± 0.64^a^	1.24 ± 0.14^a^	0.54 ± 0.01^b^	0.42 ± 0.05^b^
KSK-434	0.92 ± 0.18^b^	9.15 ± 2.70^a^	6.75 ± 0.91^a^	10.37 ± 0.18^c^	0.83 ± 0.05^b^	0.39 ± 0.03^c^	0.24 ± 0.05^c^
KS-282	0.79 ± 0.14^b^	3.31 ± 0.37^bc^	6.82 ± 0.94^a^	11.77 ± 1.57^b^	0.57 ± 0.22^c^	0.39 ± 0.05^c^	0.37 ± 0.10^b^
IR-6	0.33 ± 0.10^c^	3.37 ± 0.97^b^	4.65 ± 0.85^b^	9.46 ± 0.34 ^cd^	0.24 ± 0.03^d^	0.20 ± 0.03^d^	0.14 ± 0.03^c^
LSD_0.05_	0.27	0.73	1.24	1.38	0.15	0.07	0.13
**Young leaf tip (mg plant**^−**1**^**)**							
Basmati-2000	0.50 ± 0.06^a^	0.57 ± 0.02^c^	3.74 ± 0.09^bc^	1.74 ± 0.43^b^	0.33 ± 0.10^a^	0.39 ± 0.04^c^	0.55 ± 0.06^c^
Super Basmati	0.13 ± 0.05 ^cd^	0.52 ± 0.33^c^	3.51 ± 0.06^c^	1.75 ± 0.23^b^	0.19 ± 0.06^b^	0.25 ± 0.02^d^	0.35 ± 0.06^d^
PS-2	0.27 ± 0.09^b^	0.77 ± 0.07^b^	4.42 ± 0.45^ab^	3.83 ± 0.57^a^	0.34 ± 0.07^a^	0.69 ± 0.11^a^	0.87 ± 0.12^a^
KSK-434	0.21 ± 0.03^bc^	1.18 ± 0.32^a^	4.57 ± 0.44^a^	3.92 ± 0.75^a^	0.23 ± 0.08^b^	0.44 ± 0.03^bc^	0.68 ± 0.09^b^
KS-282	0.09 ± 0.04^d^	0.71 ± 0.23^b^	4.58 ± 0.40^a^	4.30 ± 1.82^a^	0.25 ± 0.01^b^	0.53 ± 0.16^b^	0.77 ± 0.06^ab^
IR-6	0.07 ± 0.03^d^	0.65 ± 0.17^bc^	3.13 ± 0.34^c^	1.89 ± 0.32^b^	0.11 ± 0.05^c^	0.21 ± 0.03^d^	0.30 ± 0.02^d^
LSD_0.05_	0.10	0.14	0.73	0.66	0.06	0.11	0.13
**Young leaf base (mg plant**^−**1**^**)**							
Basmati-2000	0.43 ± 0.06^a^	0.89 ± 0.14 ^cd^	4.51 ± 0.70^a^	2.22 ± 0.21^b^	0.72 ± 0.20^a^	0.74 ± 0.14^a^	0.83 ± 0.11^a^
Super Basmati	0.28 ± 0.05^bc^	1.14 ± 0.47^bc^	4.40 ± 0.61^a^	2.72 ± 0.16^ab^	0.21 ± 0.08^d^	0.33 ± 0.02^d^	0.25 ± 0.01^c^
PS-2	0.42 ± 0.10^a^	0.83 ± 0.12^d^	4.13 ± 0.13^ab^	3.50 ± 0.65^a^	0.36 ± 0.06^bc^	0.55 ± 0.05^b^	0.43 ± 0.07^b^
KSK-434	0.37 ± 0.04^ab^	2.30 ± 0.56^a^	4.24 ± 0.57^a^	2.36 ± 0.22^ab^	0.42 ± 0.06^b^	0.51 ± 0.07^b^	0.44 ± 0.06^b^
KS-282	0.19 ± 0.05^c^	0.95 ± 0.28 ^cd^	3.17 ± 0.36^b^	2.20 ± 0.57^b^	0.28 ± 0.07 ^cd^	0.47 ± 0.07^bc^	0.35 ± 0.06^bc^
IR-6	0.18 ± 0.04^c^	1.34 ± 0.34^b^	3.82 ± 0.17^ab^	2.63 ± 0.21^ab^	0.31 ± 0.11 ^cd^	0.39 ± 0.09 ^cd^	0.30 ± 0.10^bc^
LSD_0.05_	0.12	0.26	1.04	1.19	0.10	0.11	0.16
**Mature leaf tip (mg plant**^−**1**^**)**							
Basmati-2000	0.93 ± 0.15^a^	0.51 ± 0.03^e^	0.44 ± 0.08^d^	1.39 ± 0.12^b^	0.48 ± 0.05^a^	0.47 ± 0.06^a^	0.35 ± 0.02^b^
Super Basmati	0.16 ± 0.03^d^	1.02 ± 0.41^b^	0.78 ± 0.19^c^	1.86 ± 0.12^ab^	0.18 ± 0.04^c^	0.14 ± 0.01^d^	0.17 ± 0.03^d^
PS-2	0.44 ± 0.11^b^	0.69 ± 0.19 ^cd^	1.19 ± 0.08^b^	2.09 ± 0.45^a^	0.55 ± 0.12^a^	0.41 ± 0.11^b^	0.51 ± 0.07^a^
KSK-434	0.36 ± 0.07^bc^	1.47 ± 0.13^a^	1.60 ± 0.26^a^	2.09 ± 0.25^a^	0.48 ± 0.04^a^	0.37 ± 0.05^b^	0.33 ± 0.03^bc^
KS-282	0.31 ± 0.08^bcd^	0.61 ± 0.09^de^	1.32 ± 0.22^b^	1.95 ± 0.40^ab^	0.35 ± 0.09^b^	0.23 ± 0.04^c^	0.24 ± 0.02 ^cd^
IR-6	0.26 ± 0.05 ^cd^	0.76 ± 0.19^c^	0.89 ± 0.28^c^	1.48 ± 0.29^b^	0.27 ± 0.13^bc^	0.16 ± 0.03^d^	0.22 ± 0.08 ^cd^
LSD_0.05_	0.16	0.13	0.28	0.41	0.13	0.05	0.11
**Mature leaf base (mg plant**^−1^**)**							
Basmati-2000	0.55 ± 0.04^a^	0.75 ± 0.11 ^cd^	0.58 ± 0.17^d^	1.19 ± 0.13^d^	0.58 ± 0.14^a^	0.36 ± 0.04^b^	0.28 ± 0.02^a^
Super Basmati	0.20 ± 0.08^d^	1.24 ± 0.43^b^	1.10 ± 0.45^c^	2.29 ± 0.10^abc^	0.23 ± 0.02^c^	0.15 ± 0.04^de^	0.07 ± 0.01^c^
PS-2	0.44 ± 0.14^b^	0.99 ± 0.11^bc^	1.71 ± 0.12^b^	2.76 ± 0.21^ab^	0.42 ± 0.04^b^	0.50 ± 0.06^a^	0.30 ± 0.04^a^
KSK-434	0.36 ± 0.08^bc^	2.20 ± 0.69^a^	2.30 ± 0.13^a^	2.86 ± 0.40^a^	0.29 ± 0.07^c^	0.23 ± 0.02^c^	0.18 ± 0.03^b^
KS-282	0.35 ± 0.05^bc^	0.66 ± 0.09^d^	1.57 ± 0.40^b^	2.06 ± 0.39^bc^	0.39 ± 0.08^b^	0.21 ± 0.01 ^cd^	0.12 ± 0.02^bc^
IR-6	0.24 ± 0.05 ^cd^	1.00 ± 0.24^bc^	1.01 ± 0.24^c^	1.59 ± 0.08 ^cd^	0.13 ± 0.02^d^	0.09 ± 0.02^e^	0.08 ± 0.02^c^
LSD_0.05_	0.14	0.29	0.39	0.79	0.08	0.07	0.08
**Whole plant (mg plant**^−**1**^**)**							
Basmati-2000	3.96 ± 0.14^a^	6.69 ± 0.81^c^	13.25 ± 0.51^d^	19.77 ± 1.09^c^	3.97 ± 0.24^a^	3.99 ± 0.64^a^	4.01 ± 0.40^a^
Super Basmati	1.38 ± 0.28^d^	8.08 ± 0.72^c^	15.36 ± 2.47^c^	17.43 ± 0.91^c^	1.40 ± 0.09^d^	1.44 ± 0.08^d^	1.46 ± 0.09^c^
PS-2	4.07 ± 0.42^a^	11.23 ± 0.61^b^	23.20 ± 0.64^a^	32.92 ± 1.18^a^	4.08 ± 0.17^a^	4.10 ± 0.23^a^	4.11 ± 0.56^a^
KSK-434	2.83 ± 0.32^b^	19.19 ± 0.97^a^	23.52 ± 1.96^a^	26.84 ± 1.46^b^	2.86 ± 0.11^b^	2.87 ± 0.14^b^	2.88 ± 0.09^b^
KS-282	2.15 ± 0.28^c^	7.44 ± 0.44^c^	20.23 ± 1.90^b^	28.19 ± 3.26^b^	2.18 ± 0.40^c^	2.19 ± 0.12^c^	2.21 ± 0.25^bc^
IR-6	1.32 ± 0.21^d^	7.71 ± 0.24^c^	15.40 ± 2.38^c^	18.54 ± 1.06^c^	1.34 ± 0.16^d^	1.35 ± 0.07^d^	1.36 ± 0.13^c^
LSD_0.05_	0.46	1.63	1.89	3.23	0.48	0.41	0.95

Initially, plants were grown for 20 days with adequate P (200 µM Pi). At 20 DAT, three replications of each cultivar were harvested, and the remaining plants were divided into two groups receiving adequate P or no P and harvested at 30, 40, and 50 DAT. Data are shown as means ± standard error (mean ± SE, n = 3). Means sharing identical letter(s) in the same column indicate non-significant differences among cultivars at each harvest (LSD test, *P* ≤ 0.05).

#### Internal remobilization and utilization efficiency of acquired P by rice cultivars

**Figure 1:**
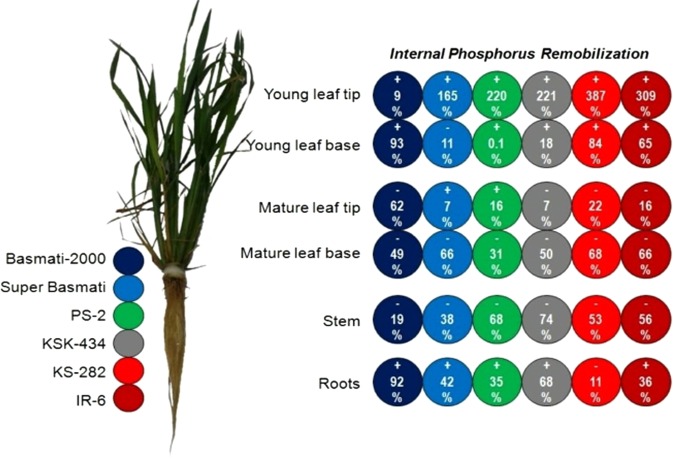
Internal phosphorus remobilization among various plant tissues of six rice cultivars at 50 days of transplanting after feeding plants for 20 days with adequate P level (200 µM Pi) and subsequent P omission for next 30 days. Positive signs indicate P accumulation while negative signs illustrate P remobilization in specific plant tissues/organs in response to P omission.

**Figure 2:**
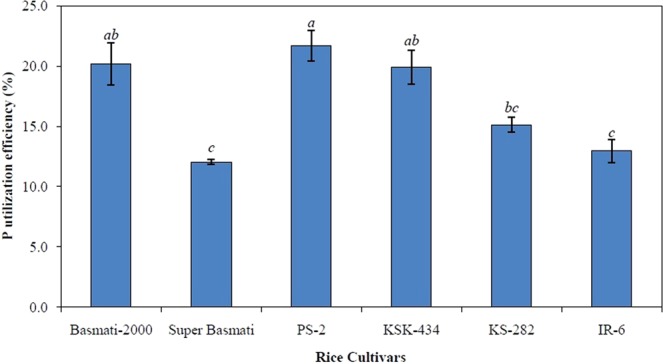
Phosphorus utilization efficiency (PUTE) of six rice cultivars calculated at two P levels under hydroponic conditions (Experiment 1). Values are means of three replicates (n = 3) and presented with standard error. Bars not sharing identical letter(s) are significantly different from each other (LSD test, *P* ≤ 0.05). LSD value for PUTE at *P* ≤ 0.05 is 5.53.

The internal P remobilization (IPR) was calculated among various plant tissues at 50 days of transplanting after feeding plants for 20 days with adequate P and subsequent P omission for next 30 days (Fig. 1). At this time, P had accumulated in the roots and young leaves after remobilization from the stem and mature leaves. Cultivars KSK-434 and PS-2 remobilized the most stem P reserves (74 and 68%, respectively) after P omission. The lowest IPR from stems occurred in Basmati-2000, but its roots accumulated the most P (92%) during P omission. Among young leaves, the apical section (tip) accumulated comparatively more P reserves than the basal section in all cultivars except Super Basmati where P contents declined upto 11% for the young leaf base. Nevertheless, basal sections of mature leaves remobilized more P than the mature leaf apical (tip) section in all cultivars except PS-2 and Super Basmati where P accumulation (16 and 7%, respectively) occurred in the mature leaf tip. Phosphorus utilization efficiency (PUTE) varied from 12.1% (Super Basmati) to 21.7% (PS-2) with a mean value of 16.9% (Fig. 2).

### Experiment 2

Two rice cultivars contrasting in P utilization efficiency - Super Basmati (P-inefficient) and PS-2 (P-efficient) - were selected from Experiment 1 and grown at two P levels (0 and 50 mg P kg^−1^) in soil-filled pots to maturity to investigate their response for paddy production, P acquisition and utilization efficiencies.

#### Yield and related attributes

**Figure 3:**
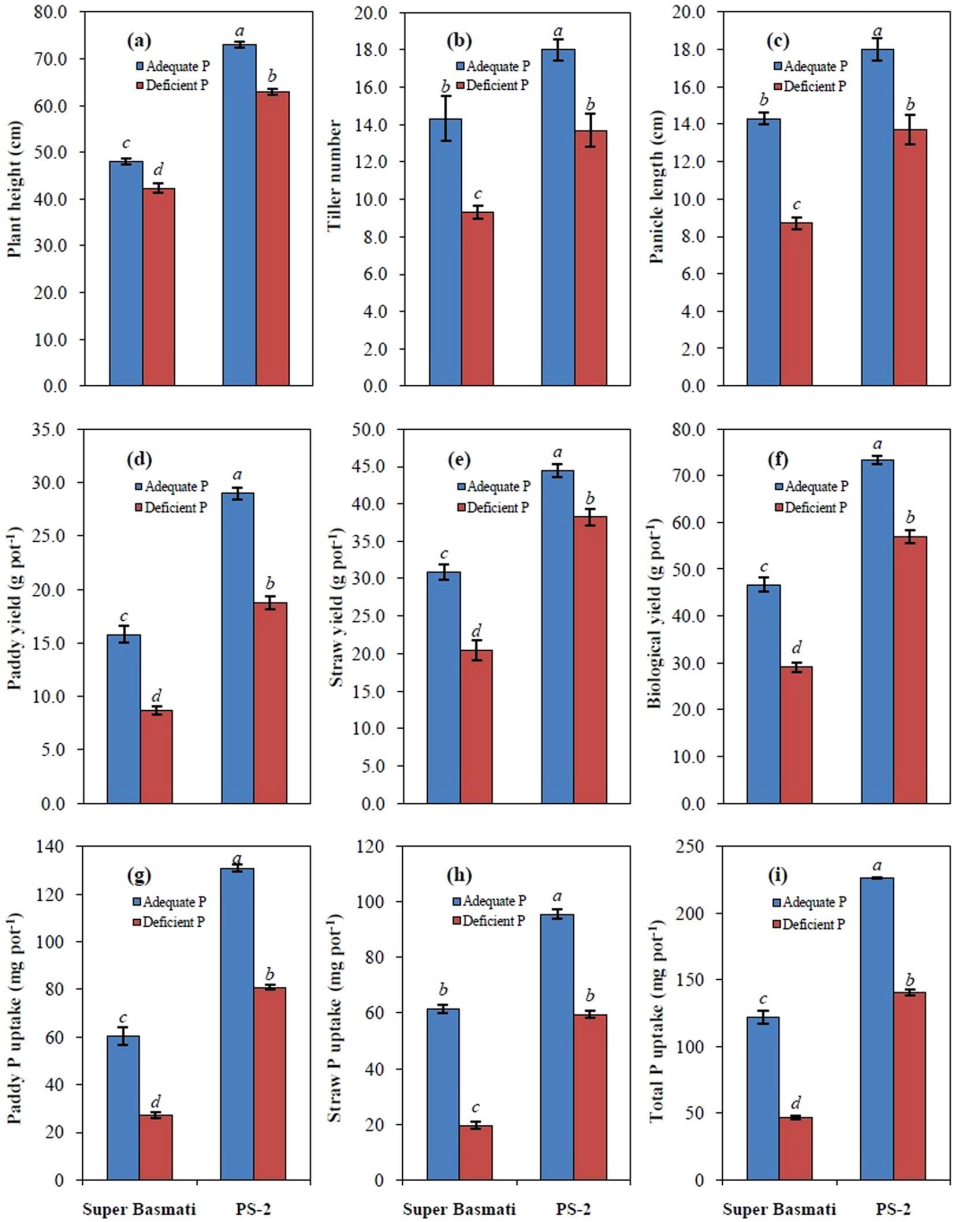
Plant height **(a)**, tiller number **(b)**, panicle length **(c)**, paddy yield **(d)**, straw yield **(e)**, biological yield **(f)**, paddy P uptake **(g)**, straw P uptake **(h)**, and total P uptake **(i)** of two rice cultivars selected from Experiment 1. Values are means of three replicates (n = 3) and presented with standard error. Bars not sharing identical letter(s) are significantly different from each other at two P levels (LSD test, *P* ≤ 0.05).

Phosphorus deficiency significantly (*P* ≤ 0.05) reduced rice yield and related attributes, i.e., plant height, tiller number and panicle length, in both rice cultivars (Table 5, Fig. 3). However, the magnitude of reduction was higher in cultivar Super Basmati than cultivar PS-2. Plant height of Super Basmati and PS-2 varied from 42.3–48.0 cm and 63.0–73.0 cm under deficient and adequate P supply, respectively. Super Basmati had more tillers under P deficiency than PS-2 (13.7 vs. 9.3). The panicle length of Super Basmati and PS-2 increased from 8.7 to 14.3 cm and 13.7 to 18.0 cm in response to deficient and adequate P, respectively. PS-2 produced the highest paddy yield (18.8 vs. 29.0 g pot^−1^) as compared to Super Basmati (8.7 vs. 15.8 g pot^−1^) in unfertilized and P-fertilized plants, respectively. Straw yield in PS-2 and Super Basmati declined in unfertilized plants (38.2 and 20.5 g pot^−1^) and increased in response to P fertilization (44.4 and 30.9 g pot^−1^), respectively. The biological yield (straw + paddy) of PS-2 and Super Basmati was 73.4 and 46.7 g pot^−1^ in P-fertilized plants and 57.0 and 29.2 g pot^−1^ in P-deficient plants, respectively.

**Table 5:** Analysis of variance for the combined effect of phosphorus level and rice cultivar on various growth parameters of soil-grown rice plants.

Source of variation	DF	PH	NTP	PL	PY	SY	BY	PPU	SPU	TPU
Phosphorus levels (P)	1	184.08***	65.33***	75.00***	223.60***	205.84***	858.52***	5160.5***	4564.6***	19431.9***
Cultivars (C)	1	1564.08***	48.00**	56.33***	406.00***	731.64***	2227.69***	11561.2***	4084.4***	29390.1***
P × C	1	14.08*	0.33 ns	1.33 ns	7.68*	13.44*	0.80 ns	214.6**	26.1*	91.1*
Error	8	1.58	2.00	0.87	1.11	3.43	4.46	13.4	6.4	24.8
Total	11									

DF = degrees of freedom; PH = plant height; NTP = tiller number per pot; PL = panicle length; PY = paddy yield; SY = straw yield; BY = biological yield; PPU = paddy P uptake; SPU = straw P uptake; TPU = total (paddy + straw) P uptake.

***significant at *P* ≤ 0.001; **significant at *P* ≤ 0.01; *significant at *P* ≤ 0.05; ns = non-significant at *P* ≥ 0.05.

#### Phosphorus uptake and efficiency relations of rice cultivars

Phosphorus uptake in paddy, straw, and total (paddy + straw) by rice cultivars grown under soil conditions with deficient and adequate P supply is depicted in Fig. 3. The rice cultivars and P levels had significant (*P* ≤ 0.05) main and interactive effects on P uptake by paddy and straw (Table 5). Average P uptake by both straw and paddy was about two-fold higher in plants with adequate P supply than deficient levels. The paddy P uptake in PS-2 and Super Basmati was 130.81 and 60.27 mg pot^−1^ under adequate P and 80.87 and 27.25 mg pot^−1^ under P deficiency, respectively. Under adequate P, straw P uptake in PS-2 and Super Basmati was 95.50 and 61.54 mg pot^−1^ and 59.44 and 19.59 mg pot^−1^ under P deficiency, respectively. The values for total P uptake ranged from 140.31 to 226.30 mg pot^−1^ for PS-2 and 46.84 to 121.81 mg pot^−1^ for Super Basmati under deficient and adequate P, respectively.

**Figure 4:**
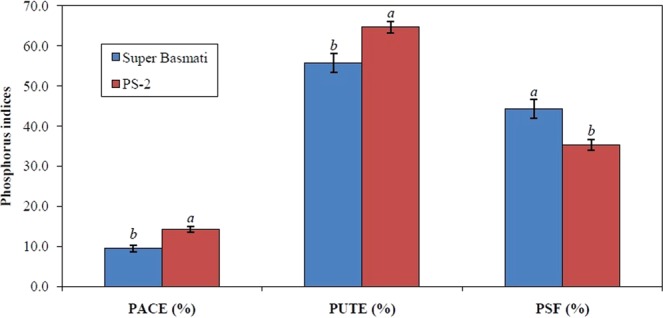
Phosphorus acquisition efficiency (PACE), phosphorus utilization efficiency (PUTE), and phosphorus stress factor (PSF) of two rice cultivars grown under soil conditions (Experiment 2). Values are means of three replicates (n = 3) and presented with standard error. For each parameter, bars not sharing identical letter(s) are significantly different from each other (LSD test, *P* ≤ 0.05). LSD values for PACE, PUTE, and PSF at *P* ≤ 0.05 are 2.88, 7.22, and 8.73, respectively.

Both rice cultivars responded differently for P-efficiency relations – P acquisition efficiency (PACE), P utilization efficiency (PUTE) and P stress factor (PSF) – at the two soil P levels (Fig. 4). The rice cultivar PS-2 had higher values than Super Basmati for PACE (14.3 vs. 9.4%) and PUTE (64.7 vs. 55.8%). The PSF describes the percent reduction in paddy production in response to P deficiency in the rooting medium. It distinguishes between P-responsive and non-responsive genotypes and elucidates the capacity of a genotype to produce biomass with P addition. Super Basmati had a larger PSF (44.2%) than PS-2 (35.3%).

## Discussion

Limited P availability to plants on arable land is a major nutritional constraint hindering plant growth and crop yields. Significant variation for P absorption and utilization among crop species and even genotypes within the same species is well documented^4,5,10,30,31^. The selection/identification of cultivars that can absorb and use P efficiently is a promising strategy to cope with environments deficient in bio-available P^12^. Phosphorus is a highly mobile element within plants that is readily remobilized/re-translocated from metabolically inactive to active sites to sustain plant growth under P deficiency^32^. In this study, we investigated the hypothesis that variation in P acquisition and remobilization is due to adaptive mechanisms that cause differential P utilization in rice cultivars.

In Experiment 1, root and shoot dry matter (DM) production under P deficiency varied between rice cultivars. Dry matter production under low-P supply is an excellent parameter for screening germplasm for higher yields and P efficiency^8,33^. Two efficient cultivars, PS-2 and Basmati-2000, produced more DM than Super Basmati and IR-6 under P starvation, which can be attributed to an increased rate of P remobilization to growing sites, i.e., young leaves and roots. Remobilization of stored P in the stem and older leaves to metabolically active sites may supplement the restricted P supply under P deficiency^13,34,35^. This was confirmed at all harvests by the differential P redistribution and accumulation in various plant tissues during P omission (Fig. 1, Table 4). Variations in shoot biomass under varying levels of P among different rice cultivars have been reported^36^. Roots are critical for plant growth and are directly exposed to the soil environment. As P mobility in soil is limited, higher plant root growth and changes in root morphology is helpful for more P uptake. Rice cultivar PS-2 had the deepest roots and the most root dry matter when exposed to P deficiency (Table 1). Under P starvation, root length and root hair density significantly increased^37^ for better P absorption. The findings of Bates and Lynch^38^ suggested that increased root growth is associated with improved plant performance under low P by exploring a larger volume of soil. The root:shoot ratio (RSR) is a further indication of the partitioned plant growth into roots and shoots to differential P supply. The RSR increased in all tested cultivars under P stress while KSK-434 had the maximum RSR. Generally, under low-P supply, plant growth is suppressed. However, the relative effect is most dominant in the case of shoot growth. Consequently, RSR increases significantly in low-P environments^39^ and is an excellent index for partitioning photosynthesized carbon between above – and below – ground plant parts. Root density and RSR generally increased under P deficiency, thus favoring P acquisition by plants^13,40^. Plants often allocate a greater portion of their biomass to the roots in response to P deficient environment. Such acclamatory response is a consequence of metabolic changes in shoot and an adjustment of carbohydrate transport to the root. P deficiency alter root-to-shoot biomass ratio by accumulation of carbohydrates in leaves and roots^41^. These findings were confirmed in the current study.

Increased remobilization of absorbed P among various tissues within plants under P deficiency might be a mechanism for better P efficiency in cultivars differing in P acquisition and utilization^32,42^. In Experiment 1, plants were grown for 20 days with adequate P (200 µM Pi), after which P was omitted from the solution for the next 30 days. As plants were unable to uptake more P from the medium, they had to remobilize previously acquired P from various plant tissues. Molecular mechanisms involved in mobilizing P from different plant tissues/organs are well reported^15,43–45^. Organic pools of P in plant tissues mainly exist as nucleic acids, phospholipids, phosphorylated proteins and P-ester metabolites^15^. Under P deficiency, phospholipids have greater potential to serve as an alternate source of P which remobilizes towards young growing plant tissues^46^. This phenomenon of replacing phospholipids in cell membranes with sulfolipids and galactolipids is known as membrane-lipid remodeling^43^. Lipid remodeling occurs both in roots and shoots of P-efficient rice genotypes under low-P conditions resulting in enhanced PUE^44^. Ribonucleases and intracellular phosphatases had crucial role in determining the amount of P to be remobilized from older to young green leaves and developing grains^45^.

During P omission, P accumulated in the roots and young leaves after remobilization from the stem and mature leaves (Fig. 1, Table 4). Cultivars KSK-434 and PS-2 remobilized the most stem P reserves (74 and 68%, respectively) after P omission. Within young leaves, the apical section (tip) accumulated comparatively more P than the basal section (Table 4). Based on differential P contents in various plant tissues during P omission; it could be assumed that P remobilization from the metabolically inactive pool to the active pool was initially more intensive in mature leaves, stem, roots, and young leaves. With persistent P stress, the stems recorded the maximum reduction in P contents. An increase or even maintenance of root P content during P stress may be related to greater allocation of assimilates to roots^47^. The higher P contents in roots and young leaves of rice under adequate P illustrates that young tissues have better storage capacity than older tissues^17^. Moreover, enhanced metabolic activities of young tissues make them stronger sinks for the already absorbed P. After removing P from the nutrient solution, plant growth may occur due to remobilization and utilization of absorbed P^48^. According to Martinez *et al*.^49^, young leaves and roots were the major sinks of stored P in soybean cultivars. Under moderate P deficiency, the transfer of P increased in the shoots of potato plants, while severely stressed plants retained P in the roots^50^. Although, more P acquisition form P-deficient medium is necessary for tolerance of a genotype to low P, however efficient remobilization of acquired P to different growing tissues can make a genotype well adapted to low-P environment^51^. Efficient internal utilization of P is generally attributed because of high photosynthetic activity per unit of P and more efficient P remobilization from older to young leaves^52^. Moreover, higher remobilization of P from older leaves to young green leaves in P-efficient rice genotypes is important to produce more photosynthates which ultimately contribute for biomass/yield production^53^.

In Experiment 2, two rice cultivars selected from the hydroponic study – Super Basmati (P-inefficient) and PS-2 (P-efficient) – were grown in soil to maturity to investigate their response for paddy production, P acquisition, and utilization efficiencies. It was clear from the results that the high-yielding cultivar PS-2 had more paddy P uptake and the low-yielding cultivar Super Basmati had the lowest paddy P uptake under both P levels. Fageria^54^ also reported increased P uptake in grains of high-yielding rice genotypes at low P. According to Baligar *et al*.^55^, more P uptake in plants grown at high P compared with those at low P is due to an increased root fine hair density. The rice cultivars varied in P-efficiency relations (PACE, PUTE, and PSF) under P stress. The high-yielding PS-2 had greater PACE and PUTE than the low-yielding Super Basmati. Phosphorus utilization and uptake efficiencies are important for selecting genotypes under P-deficiency stress. The P-use efficiency of cultivars is associated with the amount of dry matter produced per unit of absorbed P^56^. The variation between cultivars for PUE might be due to differential P absorption characteristics or distribution patterns within plants. Both cultivars varied in their relative reduction in paddy yield due to P deficiency (PSF). The PSF value distinguishes between P-responsive and non-responsive cultivars and the comparative ability of a cultivar to produce biomass with P addition^4,5,13^. Cultivar PS-2 had a lower PSF value than Super Basmati under P stress (35% vs. 44%). Under P deficiency, PS-2 produced more paddy yield than Super Basmati and responded well to P application. Super Basmati was less desirable due to poor performance under varying P supply. PS-2 also had higher P uptake than Super Basmati, indicating an important contributing trait to its high P-efficiency. The lower P-efficiency of Super Basmati was further supported by its higher PSF value than PS-2. Fageria *et al*.^30^ reported variation in the abilities of lowland rice cultivars to absorb P, and its remobilization and efficient utilization overtime. Plant traits associated with efficient P acquisition and internal utilization are heritable and can be exploited more extensively to develop P-efficient cultivars^27^.

## Conclusions

Significant genetic variation exists among the tested rice cultivars for P efficiency. Rice cultivars with higher P uptake, acquisition efficiency, utilization efficiency and lower PSF values were efficient (low-P tolerant) and thus more desirable for adaptation to soils low in available P. The cultivars with high biomass/paddy yield at both P levels (PS-2 and Basmati-2000) have better internal remobilization of absorbed P and photosynthate translocation to young/active plant tissues. Hence, P partitioning and biomass allocation to roots should be considered when screening germplasm in P-efficiency programs.

## Materials and Methods

### Experiment 1

#### Plant material, growth conditions, and management

Seeds of six rice cultivars (Basmati-2000, Super Basmati, PS-2, KSK-434, KS-282 and IR-6) were kindly provided by the Rice Research Institute, Kala Shah Kaku, Pakistan. Seeds were surface sterilized with 3% sodium hypochlorite solution and sown in polyethylene foil-lined metal trays containing two inches of washed riverbed sand. Distilled water was used to maintain optimum moisture for seed germination and seedling establishment. Two weeks after seed germination, the root systems of seedlings were carefully rinsed in distilled water to remove any adhering sand. Uniform seedlings were transferred to 25 L plastic tubs containing modified Johnson’s nutrient solution in a completely randomized design. The composition of the full-strength nutrient solution (pH 5.5) was 5.0 mM N, 3.5 mM K, 1.5 mM Ca, 0.5 mM Mg, 2.05 mM S, 50 µM Cl, 0.5 µM Mo, 25.0 µM B, 0.5 µM Cu, 2.0 µM Mn, 2.0 µM Zn, and 50.0 µM Fe as Fe-EDTA. The seedlings were held with foam plugs in the holes of a thermopore sheet placed at the top of each tub. Initially, plants grew for 20 days after transplanting (DAT) with adequate P supply (200 µM Pi). At 20 DAT, three replications of each cultivar were harvested, and the remaining plants were divided into two groups that received nutrient solution with either 200 µM Pi or without Pi and harvested at 30, 40 and 50 DAT. At each harvest, plant roots were rinsed (2–3 times) with distilled water to remove the nutrient solution. Shoot samples were washed with distilled water, blotted dry, and then separated into the stem, young (top) leaves and mature (lower) leaves. Young and mature leaves were further dissected from the middle into leaf tip (upper half) and leaf base (lower half). Plant material was dried at 70 °C for 48 h in a forced air-driven oven and stored under desiccation until weighing for dry biomass.

### Experiment 2

#### Experimental site, design and crop management

Two rice cultivars contrasting in P utilization efficiency were selected from Experiment 1 [Super Basmati (P-inefficient) and PS-2 (P-efficient)] and grown in plastic pots containing 7 kg of soil. Bulk soil from the top 15 cm was collected from the research area of the Institute of Soil and Environmental Sciences, University of Agriculture, Faisalabad, Pakistan. A composite sample of collected soil was subjected to various physicochemical properties. In brief, the soil was a clay loam, non-saline (EC_e_, 2.12 dS m^−1^), alkaline in soil reaction (pH_s_, 7.76), low in Kjeldahl nitrogen (0.04%), organic matter (0.71%) and available phosphorus (2.01 mg kg^−1^) and high in available potassium (160 mg kg^−1^). The experiment was arranged in a completely randomized factorial design having three replicates. All pots were irrigated with canal water, applied from the top to soil saturation and kept for two days for soil settling. The required amounts of P and K were added at the time of pot filling while N was applied in three equal splits. Both rice cultivars were grown at two P levels [i.e., adequate (P addition at 50 mg kg^−1^ soil) and deficient (native soil P at 5.30 mg kg^−1^ without external P addition)]. Seeds of rice cultivars were sown in nursery trays containing similar soil to the pots. At the three-leaf stage, three uniform and healthy seedlings of each cultivar were transplanted to each pot. A water layer (1–2 cm) on the soil surface was maintained during the entire crop period. At maturity, plants were harvested and threshed manually to separate paddy from straw. After recording yield and related attributes, plant material was oven dried at 70 °C till further analysis.

### Soil analysis

In order to determine physicochemical properties of soil used in experiment 2, soil sample was air-dried and grounded to pass through a 2 mm sieve. Soil texture was determined by performing mechanical analysis of soil separates (sand, silt, and clay) using hydrometer method by dispersing soil in sodium hexametaphosphate solution^57^. Soil reaction of the saturated soil paste (pH_s_) and electrical conductivity of saturation extract (EC_e_) were determined with pH and EC meter, respectively^58^. Soil available phosphorus and potassium were estimated using ammonium bicarbonate-diethylene triamine penta-acetic acid (AB-DTPA) as extracting solution^59^. Kjeldahl nitrogen was determined following the method described by Jackson^60^. Soil organic matter (SOM) was quantified by chromic acid digestion according to Walkley-Black method^61^.

### Plant analysis

Oven-dried plant material from both experiments was finely ground in a sample grinder (IKA Werke, Wilmington, USA). Ground samples (0.3 g each) were wet digested using 10 mL of di-acid digestion mixture [(HNO_3_:HClO_4_ (5:1, *v/v*)]. Total P concentration in plant samples was determined following the vanadate–molybdate method^62^ using UV-visible spectrophotometer (Shimadzu UV-VIS 1201, Shimadzu Co. Kyoto, Japan).

### Calculation methods

The following P-efficiency relations were calculated to establish the relationship between P levels and plant growth of rice cultivars, i.e., P uptake^63^, internal P remobilization^64^, acquisition efficiency^2^, utilization efficiency^65^, and stress factor^66^ using the following formulas:

- Phosphorus uptake (PU)
  $${\rm{PU}}({\rm{mg}}\,{{\rm{plant}}}^{-1})={\rm{P}}\,{\rm{concentration}}({\rm{mg}}\,{{\rm{g}}}^{-1})\times {\rm{dry}}\,\text{matter}(g\,{{\rm{plant}}}^{-1})$$
- Internal phosphorus remobilization (IPR)
  $${\rm{IPR}}\,( \% )=\frac{{\rm{P}}\,{{\rm{uptake}}}_{{\rm{before}}{\rm{P}}{\rm{omission}}}-{\rm{P}}\,{{\rm{uptake}}}_{{\rm{after}}{\rm{P}}{\rm{omission}}}}{{\rm{P}}\,{{\rm{uptake}}}_{{\rm{before}}{\rm{P}}{\rm{omission}}}}\times 100$$
- Phosphorus acquisition efficiency (PACE)
  $${\rm{PACE}}( \% )=\frac{{\rm{P}}\,{{\rm{uptake}}}_{{\rm{adequate}}{\rm{P}}}-{\rm{P}}\,{{\rm{uptake}}}_{{\rm{deficient}}{\rm{P}}}}{{\rm{Quantity}}\,{\rm{of}}\,{\rm{P}}\,{\rm{applied}}}\times 100$$
- Phosphorus utilization efficiency (PUTE)
  $${\rm{PUTE}}( \% )=\frac{{\rm{Paddy}}\,{{\rm{yield}}}_{{\rm{deficient}}{\rm{P}}}}{{\rm{Paddy}}\,{{\rm{yield}}}_{{\rm{adequate}}{\rm{P}}}}\times 100$$
- Phosphorus stress factor (PSF)

$${\rm{PSF}}( \% )=\frac{{\rm{Paddy}}\,{{\rm{yield}}}_{{\rm{adequate}}{\rm{P}}}-{\rm{Paddy}}\,{{\rm{yield}}}_{{\rm{deficient}}{\rm{P}}}}{{\rm{Paddy}}\,{{\rm{yield}}}_{{\rm{adequate}}{\rm{P}}}}\times 100$$

### Statistical analysis

The computer software STATISTIX 8.1 (Analytical Software, Inc., Tallahassee, FL, USA) was used to perform statistical analysis following the methods of Steel *et al*.^67^. All data reported in this study are the means of three replicates and presented with standard errors. First experiment was conducted following completely randomized desing and the significant differences among rice cultivars regarding growth, biomass production and P accumulation in various plant tissues were differentiated at each P level using one-way analysis of variance (ANOVA) technique with an associated least significant difference test at 5% probability level (LSD, *P* ≤ 0.05). In second experiment, completely randomized design with factorial arrangement was employed and the results for yield, P uptake and P-efficiency relations were compared by two-way ANOVA technique with an associated LSD test at *P* ≤ 0.05. The graphical presentation of data was performed usign Microsoft Office (Redmond, WA, USA).

## Data Availability

All data generated or analysed during this study are included in this published article.

## References

[1] Brady, N. C. & Weil, R. R. *The Nature and Properties of Soils* (14^th^ Ed). Prentice Hall, Upper Saddle River, New Jersey (USA 2008).

[2] Baligar V, Fageria N, He Z (2001). Nutrient use efficiency in plants. Comm. Soil Sci. Plant Analysis..

[3] Stewart WM, Dibb DW, Johnston AE, Smyth TJ (2005). The contribution of commercial fertilizer nutrients to food production. Agron. J..

[4] Irfan M, Shah JA, Abbas M (2017). Evaluating the performance of mungbean genotypes for grain yield, phosphorus accumulation and utilization efficiency. J. Plant Nutr..

[5] Abbas M, Irfan M, Shah JA, Memon MY (2018). Intra-specific variations among wheat genotypes for phosphorus use efficiency. Asian J. Agric. Biol..

[6] Irfan M, Abbas M, Shah JA, Memon MY (2018). Internal and external phosphorus requirements for optimum grain yield are associated with P-utilization efficiency of wheat cultivars. Commun. Soil Sci. Plant Anal..

[7] Dawson CJ, Hilton J (2011). Fertiliser availability in a resource-limited world: production and recycling of nitrogen and phosphorus. Food Pol..

[8] Abbas M, Irfan M, Shah JA (2018). Differential performance of wheat genotypes for grain yield, phosphorus uptake and utilization at low and high phosphorus levels. Proc. Pak. Acad. Sci.: B. Life Environ. Sci..

[9] Ozturk L, Eker S, Torun B, Cakmak I (2005). Variation in phosphorus efficiency among 73 bread and durum wheat genotypes grown in a phosphorus-deficient soil. Agron. J..

[10] Aziz T, Finnegan PM, Lambers H, Jost R (2014). Organ‐specific phosphorus‐allocation patterns and transcript profiles linked to phosphorus efficiency in two contrasting wheat genotypes. Plant, Cell Environ..

[11] Irfan M (2019). Differential performance of lowland rice cultivars for phosphorus uptake and utilization efficiency under hydroponic and soil conditions. Intr. J. Agric. Biol..

[12] Aziz T, Steffens D, Rahmatullah, Schubert S (2011). Variation in phosphorus efficiency among *Brassica* cultivars II: Changes in root morphology and carboxylate exudation. J. Plant Nutr..

[13] Aziz T, Ahmed I, Farooq M, Maqsood MA, Sabir M (2011). Variation in phosphorus efficiency among *Brassica* cultivars I: Internal utilization and phosphorus remobilization. J. Plant Nutr..

[14] Pearse SJ, Veneklaas EJ, Cawthray GR, Bolland MDA, Lambers H (2006). Carboxylate release of wheat, canola and 11 grain legume species as affected by phosphorus status. Plant Soil..

[15] Veneklaas EJ (2012). Opportunities for improving phosphorus-use efficiency in crop plants. New Phytol..

[16] Shen J, Li H, Neumann G, Zhang F (2005). Nutrient uptake, cluster root formation and exudation of protons and citrate in *Lupinus albus* as affected by localized supply of phosphorus in a split-root system. Plant Sci..

[17] Abel S, Ticconi CA, Delatorre CA (2002). Phosphate sensing in higher plants. Physiol. Plant..

[18] Abbas M, Shah JA, Irfan M, Memon MY (2018). Remobilization and utilization of phosphorus in wheat cultivars under induced phosphorus deficiency. J. Plant Nutr..

[19] Irfan M (2019). Biomass and phosphorus accumulation, partitioning and remobilization during grain development in wheat under phosphorus deficiency. Intr. J. Agric. Biol..

[20] Aziz T, Lambers H, Nicol D, Ryan MH (2015). Mechanisms for tolerance of very high tissue phosphorus concentrations in *Ptilotus polystachyus*. Plant, Cell Environ..

[21] Vance CP, Uhde‐Stone C, Allan DL (2003). Phosphorus acquisition and use: critical adaptations by plants for securing a nonrenewable resource. New Phytol..

[22] Manschadi AM, Kaul HP, Vollmann J, Eitzinger J, Wenzel W (2014). Developing phosphorus-efficient crop varieties: An interdisciplinary research framework. Field Crops Res..

[23] Abdallah M (2010). Effect of mineral sulphur availability on nitrogen and sulphur uptake and remobilization during the vegetative growth of *Brassica napus* L. J. Exp. Bot..

[24] Malagoli P, Laine P, Rossato L, Ourry A (2005). Dynamics of nitrogen uptake and mobilization in field-grown winter oil seed rape (*Brassica napus* L.) from stem extension to harvest: I. Global N flows between vegetative and reproductive tissues in relation to leaf fall and their residual N. Annals Bot..

[25] Avice J-C, Etienne P (2014). Leaf senescence and nitrogen remobilization efficiency in oil seed rape (*Brassica napus* L.). J. Exp. Bot..

[26] White, P. J. Long-distance transport in the xylem and phloem. In: Marschner, P. (ed) Marschner’s Mineral Nutrition of Higher Plants (3^rd^ ed.) Elsevier, Berlin, pp. 49–70 (2012).

[27] Yaseen M, Malhi SS (2009). Variation in yield, phosphorus uptake, and physiological efficiency of wheat genotypes at adequate and stress phosphorus levels in soil. Commun. Soil Sci. Plant Anal..

[28] Rose TJ, Pariasca-Tanaka J, Rose MT, Fukuta Y, Wissuwa M (2010). Genotypic variation in grain phosphorus concentration, and opportunities to improve P-use efficiency in rice. Field Crops Res..

[29] Vellaikumar S, Malarvizhi P (2017). Phosphorus use efficiency of selected rice varieties. Trends Biosci..

[30] Fageria NK, Santos AB, Heinemann AB (2011). Lowland rice genotypes evaluation for phosphorus use efficiency in tropical lowland. J. Plant Nutr..

[31] Akhtar MS, Oki Y, Nakashima Y, Adachi T, Nishigaki M (2016). Phosphorus stress-induced differential growth, and phosphorus acquisition and use efficiency by spring wheat cultivars. Commun. Soil Sci. Plant Analysis..

[32] Akhtar MS, Oki Y, Adachi T (2008). Intra-specific variations of phosphorus absorption and remobilization, P forms, and their internal buffering in *Brassica* cultivars exposed to a P‐stressed environment. J. Integ. Plant Biol..

[33] Rengel Z, Marschner P (2005). Nutrient availability and management in the rhizosphere: exploiting genotypic differences. New Phytol..

[34] Marschner, P. *Mineral nutrition of higher plants*. (2^nd^ ed.). Academic Press, (London 1995).

[35] Peng Z, Li C (2005). Transport and partitioning of phosphorus in wheat as affected by P withdrawal during flag-leaf expansion. Plant Soil..

[36] Gill MA, Mansoor S, Aziz T (2002). Rahmatullah & Akhtar, M. S. Differential growth response and phosphorus utilization efficiency of rice genotypes. Pak. J. Agric. Sci..

[37] Ma, Z., Bielenberg, D. G., Brown, K. M. & Lynch, J. P. Regulation of root hair density by phosphorus availability in *Arabidopsis thaliana*. *Plant, Cell Environ*. **24**, 459–467 (2201).

[38] Bates TR, Lynch JP (2001). Root hairs confer a competitive advantage under low P availability. Plant Soil..

[39] Lu Y, Wassmann R, Neub HU, Breno CS (1999). Impact of phosphorus supply on root exudation, aerenchyma formation, and methane emission of rice plants. Biogeochem..

[40] Fohse D, Claassen N, Jungk A (1991). Phosphorus efficiency of plants. II. Significance of root radius, root hairs and cation anion balance for phosphorus influx in seven plant species. Plant Soil..

[41] Hermans C, Hammond JP, White PJ, Verbruggen N (2006). How do plants respond to nutrient shortage by biomass allocation?. Trends Plant Sci..

[42] Lambers H, Brundrett MC, Raven JA, Hopper SD (2010). Plant mineral nutrition in ancient landscapes: High plant species diversity on infertile soils is linked to functional diversity for nutritional strategies. Plant Soil..

[43] Tjellstrom H, Andersson MX, Larsson KE, Sandelius AS (2008). Membrane phospholipids as a phosphate reserve: the dynamic nature of phospholipid-to-digalactosyl diacylglycerol exchange in higher plants. Plant Cell Environ..

[44] Mehra P, Pandey BK, Giri J (2016). Comparative morphophysiological analyses and molecular profiling reveal Pi-efficient strategies of a traditional rice genotype. Front. Plant Sci..

[45] Jeong K (2017). Phosphorus remobilization from rice flag leaves during grain filling: an RNA-seq study. Plant Biotechnol. J..

[46] Zhang Y (2019). Silicon compensates phosphorus deficit-induced growth inhibition by improving photosynthetic capacity, antioxidant potential, and nutrient homeostasis in tomato. Agronomy..

[47] Burauel P, Wieneke J, Ffihr F (1989). Carbohydrate status in roots of two soybean varieties: a possible parameter to explain different efficiencies concerning phosphate uptake. Plant Soil..

[48] Rausch C, Bucher M (2002). Molecular mechanisms of phosphate transport in plants. Planta.

[49] Martinez HEP, Novais RF, Rodrigues LA, do Sacramento LVS (2005). Phosphate forms in plant and their internal buffering in five soybean cultivars. Revista Brasil. Ciencia Solo..

[50] Cogliati DH, Clarkson DT (1983). Physiological changes and phosphate uptake by potato plants during development of and recovery from phosphate deficiency. Physiol. Plant..

[51] Wu P, Shou H, Xu G, Lian X (2013). Improvement of phosphorus efficiency in rice on the basis of understanding phosphate signaling and homeostasis. Curr Opin. Plant Biol..

[52] Stigter KA, Plaxton WC (2015). Molecular mechanisms of phosphorus metabolism and transport during leaf senescence. Plants.

[53] Assuero SG, Mollier A, Pellerin S (2004). The decrease in growth of phosphorus-deficient maize leaves is related to a lower cell production. Plant Cell Environ..

[54] Fageria NK (2014). Yield and yield components and phosphorus use efficiency of lowland rice genotypes. J. Plant Nutr..

[55] Baligar VC, Fageria NK, Elrashidi M (1998). Toxicity and nutrient constraints on root growth. Hort. Sci..

[56] Siddiqi MY, Glass ADM (1981). Utilization index: A modified approach to the estimation and comparison of nutrient utilization efficiency in plants. J. Plant Nutr..

[57] Bouyoucos GJ (1962). Hydrometer method improved for making particle size analysis of soils. Agron. J..

[58] Anderson, J. M. & Ingram, J. A. I. *Tropical soil biology and fertility*. Wallingford, UK: CAB International (1993).

[59] Soltanpour PN, Workman S (1979). Modification of the NaHCO_3_ DTPA soil test to omit carbon black. Commun. Soil Sci. Plant Anal..

[60] Jackson, M. L. *Soil chemical analysis*. p. 151–153. Englewood Cliffs, NJ: Prentice Hall Inc. (1962).

[61] Nelson, D. W. & Sommers, L. E. Total carbon, organic carbon and organic matter. In *Methods of soil analysis*, Part 2, eds. Page, A. L., Miller, R. H. & Keeney, D. R. p. 539–579, Chemical and microbiological properties. Madison, Wisconsin, American Society of Agronomy (USA 1982).

[62] Chapman, H. D. & Pratt, P. F. *Methods of analysis for soils, plantsand waters*. University of California, Division of Agricultural Science, Riverside, (USA 1961).

[63] Zhang Z (2007). Differential responses of conventional and Bt-transgenic cotton to potassium deficiency. J. Plant Nutr..

[64] Maillard A (2015). Leaf mineral nutrient remobilization during leaf senescence and modulation by nutrient deficiency. Front. Plant Sci..

[65] Gunes A, Inal A, Alpaslan M, Cakmak I, Walton PEG (2006). Genotypic variation in phosphorus efficiency between wheat cultivars grown under greenhouse and field conditions. Soil Sci. Plant Nutr..

[66] Hunt, R. *Plant growth analysis*. Edwards Arnold Publ. Ltd., (London 1978).

[67] Steel, R. G. D., Torrie, J. H. & Dicky, D. A. *Principles and Procedures of Statistics-A Biometrical Approach*. McGraw-Hill Book Inter. Co., (Singapore 1997).

